# Egg Production Systems, Open Space Allowance and Their Effects on Physical Parameters and Fatty Acid Profile in Commercial Eggs

**DOI:** 10.3390/ani11020265

**Published:** 2021-01-21

**Authors:** Stefano Paolo Marelli, Manuela Madeddu, Maria Grazia Mangiagalli, Silvia Cerolini, Luisa Zaniboni

**Affiliations:** Dipartimento di Medicina Veterinaria, Università degli Studi di Milano, Via dell’Università 6, 26900 Lodi, LO, Italy; stefano.marelli@unimi.it (S.P.M.); grazia.mangiagalli@unimi.it (M.G.M.); silvia.cerolini@unimi.it (S.C.); luisa.zaniboni@unimi.it (L.Z.)

**Keywords:** eggs, production system, welfare, quality, fatty acids

## Abstract

**Simple Summary:**

Animal welfare is a major concern in consumer’s decision about animal products. Nowadays it is possible to find eggs produced in various systems closer or not to hens’ specific behavioural requirements. In this study we compared physical characteristics and fatty acid profile (being the nutritional value of the egg intimately linked to its lipids content) in four differently labelled eggs according to the production system: enriched cage, alternative (small outdoor area), litter floor and organic (large outdoor area). We found many significant differences in both physical characteristics and fatty acids profiles as an effect of the production system and the outdoor space availability.

**Abstract:**

Food function is nowadays not merely limited to nourishment supplying; consumers’ interest is oriented to food healthiness and nutritional value, animal welfare, environmental impact of animal productions, and products’ traceability. The objective of the present work is to compare physical parameters and fatty acids profiles of market eggs produced from hens housed in four different systems. In addition, the effects of the presence of an outdoor space allowance (IND = no outdoor space allowance, OUT = outdoor space allowance) on the same parameters have been investigated. Sixty-nine market eggs from four different production systems labelled as Alternative (ALT), Enriched Cage (ECA), Litter Floor (LIF), and Organic (ORG) have been analysed. Physical parameters and fatty acid concentrations were measured. An ANOVA analysis was performed with production system and outdoor space allowance as sources of variation, two Principal Component Analyses (PCA) were carried out with physical parameters and fatty acid parameters as variables. The effects of the complex interactions occurring among production system, hen welfare, and eggs quality have been analysed in marketed eggs leading to the conclusion that eggs from different production systems available on the market are characterized by differences in fatty acid profile and physical parameters. In physical parameters the differences among systems were influenced by the whole egg weight, albumen weight and yolk weight. In fatty acids parameters the determining variables are the content in polyunsatured fatty acids (PUFA), linoleic acid concentration, and n6/n3 ratio.

## 1. Introduction

Food quality can be considered as one of the main influencing topics in public interest, policy, agriculture, industry, economics, and research. Consumers’ growing interest and criticism towards food production both at farm and at processing level is evident; animal welfare and organic production are two of the most important critical points [1]. Brunso and colleagues in 2002 reported that consumers’ perception of quality is based on four parameters: taste, health, convenience and production systems where animal welfare and organic production play a pivotal role [2]. Furthermore, animal welfare itself is considered a healthiness indicator in animal production [1]. Food products characterization is evolving moving from processing level to the inclusion of farm level differentiation; this trend follows consumers’ demand for animal welfare-oriented production systems [1].

Food function is nowadays not merely limited to nourishment supplying; consumers’ interest is oriented to food healthiness [3] and nutritional value, animal welfare, environmental impact of animal productions, and products’ traceability. The Total Quality concept defines in the third point of the list the nutritional quality of an animal product as related to the composition of proteins and lipids and the presence of macro and trace elements and the absence of allergenic compounds [4]. According to Aumaitre [5], fatty acid profile can be considered an ‘additional chemical factor’ defining the quality of animal productions. The quality of the product involves not only product characteristics but animal welfare and production systems (rearing conditions, feed, facilities, transport) also; traceability and farm assurance procedures including official certification of organic productions are powerful tools to certify these different aspects. Furthermore, sustainability of animal production concept is strictly related to the satisfaction of human and animal welfare requirements [6].

*Gallus gallus domesticus* egg can be considered the cheapest and the most common nutritionally complete food in the world; it is a rich source of lipids and amino acids [7]. Yolk fatty acids have a central role in egg nutritional properties evaluation. Egg lipids have a high biological and nutritional value: they are the major energy source and provide different essential components for maternal liver physiological reaction and embryo tissues development and functionality [8]. Egg yolk fatty acid profile variations may be linked to differences in feed composition, genetic strain, liver physiological reaction (desaturation and elongation) and polyunsatured fatty acids (PUFA) incorporation efficiency into Very Low Density Lipoprotein (VLDP) [9]. PUFAs have specific regulatory functions being involved in the synthesis of a range of biologically active compounds such as eicosanoids [7]. According to Uauy and Castillo [10] blood pressure, vasoconstriction and vasodilatation, thrombocyte aggregation, inflammatory reaction and leukocyte activity together with bronchial constriction and uterine contractility are some of the cell and tissue physiological functions regulated by these autocrine and paracrine mediators.

Welfare friendly egg production systems with their space allowance offer the birds the possibility to express their specie specific behavioural patterns; however, there is generally very low mortality in cages, and feather pecking and cannibalism are rare [11]. The importance of egg alternative production systems has grown due to the European Union legislation and particularly to the Council Directive 1999/74/EC of 19 July 1999 laying down the minimum standards for the protection of laying hens and to the Commission Directive 2002/4/EC of 30 January 2002 on the registration of establishments keeping laying hens, covered by Council Directive 1999/74/EC; layers’ unenriched cages have been prohibited from the 1st of January 2012. In addition, Council Regulation 2007/834/EC and Commission Regulation 2008/889/EC about organic production and labelling of organic products define all the requirements for organic production systems and organic product certification. Marketing standards of eggs are defined by Commission Regulation No 557/2007/EC of 23 May 2007 laying down detailed rules for implementing Council Regulation No 1028/2006/EC on marketing standards for eggs, Commission Regulation 508/2008/CE laying down detailed rules for implementing Council Regulation (EC) No 1234/2007 about standards for eggs’ marketing, Regulation (EU) No 1308/2013 of the European Parliament and of the Council of 17 December 2013 establishing a common organisation of the markets in agricultural products and repealing Council Regulations (EEC) No 922/72, (EEC) No 234/79, (EC) No 1037/2001, and (EC) No 1234/2007 which specify that Class A eggs (fresh eggs) shall also be graded by weight. Eggs grading by weight has been listed in Commission Regulation (EC) No 2295/2003 of 23 December 2003 introducing detailed rules for implementing Council Regulation (EEC) No 1907/90 on certain standards for market eggs (23/01/2006 version): XL—very large 73 g and more; L—large: from 63 g up to 73 g; M—medium: from 53 g up to 63 g; S—small: under 53 g.

Every housing system is characterized by positive and negative aspects: considering the commonly used welfare indicators: behaviour, physiology, health, production (characterized by a high number of variables). Housing system evaluation should analyse design criteria related to welfare needs (e.g., space allowance) and performance criteria as indicator of good welfare (e.g., production and physiology) [12]. There is a close link within housing system, welfare, and production, being the physiological and behavioural responses to distress an impairing effect on organism whole efficiency and, as consequence, on productive efficiency. Egg production can be affected under a quantitative and a qualitative point of view. However, links with egg nutritional characteristics and housing system and birds’ welfare are rare and contrasting.

The objective of the present work is to compare physical parameters and fatty acids profiles within market eggs from hen housed in four different systems: enriched cage, slat floor, litter floor, and organic system. In addition, the effects of the presence of an outdoor space area on the same parameters have been investigated.

## 2. Materials and Methods

### 2.1. Eggs and Production Systems

Sixty-nine brown shelled market eggs (A category, M–L) from four different production systems labelled as Alternative (ALT; N = 18), Enriched Cage (ECA; N = 18), Litter Floor (LIF; N = 15) and Organic (ORG; N = 18) have been analysed. Production systems characteristics according to European and National legislation have been listed in Table 1. All eggs were one day old and stored at 5 °C before analysis.

### 2.2. Physical Parameters

Physical parameters were recorded by individual weighing (Sartorius Analytic A200S, Goettingen, Germany) of the whole egg (WE), the yolk (YOL; separated with a yolk separation cup for cooking use), the albumen (ALB) and the shell (SHE; membranes included). Albumen weight/whole egg weight, yolk weight/whole egg weight and shell weight/whole egg weight ratios were calculated.

### 2.3. Total Lipids and Fatty Acid Profile

The total lipids (TL) were extracted from singular yolk samples in a suitable excess of chloroform/methanol (2:1, *v:v*) [13,14]. Fatty acids of TL were trans-methylated by refluxing in methanol: toluene: sulphuric acid (20:10:1, *v:v:v*) in the presence of pentadecanoic acid standard [15]. Fatty acid quantification was obtained by gas chromatography by injection via a CP9010 autosampler (Chrompack, Speck Analytical, London, UK) onto a capillary column (Carbowax, 30 m × 0.25 mm, film thickness 0.25 μm; Alltech ltd., Carnforth, UK) in a CP9001 Chrompack gas chromatograph connected to a data processing system: EZ-Chrom data handling system (Speck Analytical, UK). The identification of the peaks was determined by comparison with the retention times of external standard fatty acid methyl ester mixtures. The amount of each fatty acid was calculated comparing fatty acid peak areas to the peak area of Pentadecanoic fatty acid (standard) [16]. The proportion of total saturated (SFA), monounsaturated (MUFA) and polyunsaturated fatty acids (PUFA) and the n-6/n-3 ratio were calculated. Total Lipids (TL) were calculated as total content (g) in 100 g of edible egg (YOL + ALB).

### 2.4. Statistical Analysis

Data analysis was performed by ANOVA using General Linear Model procedure of SPSS. In our model dependent variables were eggs’ physical parameters (8 variables) and fatty acid profiles (11 variables), sources of variation were production system (System; 4 variables) and Outdoor space allowance (OSA; IND = indoor, OUT = outdoor area; 2 variables), the post hoc Bonferroni test was used to investigate the significant differences both in production systems and in the OSA groups (*p* ≤ 0.05) [17]. Two principal component analysis (PCA; variance-covariance matrix) were carried out with fatty acids (11 factors) and physical parameters (8 factors) as variables. Two scatterplots were produced, Scree plot test was used to choose principal components to explain the majority of the variation in the dataset (see additional tables for percentage of variance and PCA statistics details) [18].

## 3. Results

Results on egg physical parameters and related ratios are presented in Table 2. Whole egg weights showed slight but significant differences, the heaviest were ALT eggs. No significant differences were recorded in albumen weight. Yolk weight was significantly higher in ALT eggs. Eggshell weight was lower in ORG eggs. ALB/WEG ratio reveals a lower albumen content in ALT eggs which are characterized by a higher ratio YOL/WEG together with ORG. SHE/WEG ratio differences were not significant. TL content showed higher values in ORG and ALT eggs, on the contrary LIF eggs showed the lowest lipidic content.

Considering the effect of OSA on physical characteristics and TL yolk content, the presence of the en plein air area significantly influences the yolk weight with OUT being higher than IND with a higher ratio in YOL/WEG too in the OUT eggs. As a consequence, TL content was also higher in OUT eggs than in IND eggs.

Fatty acid composition of yolk TL in the four system groups and the two OSA groups are listed in Table 3. Production systems significantly influence mean values of all the identified fatty acids, with the only exception of docosahexaenoic acid. Significant differences are shown for SFA, MUFA, PUFA, and n6/n3 ratio. ECA eggs showed the lowest concentration of palmitic acid and ORG eggs the highest; however, the only significant difference was found between ECA and ALT eggs. The lowest proportion of stearic acid was found in LIF eggs and it was significantly different when compared to ALT and ORG eggs which had the highest concentration. Oleic acid highest concentration was recorded in LIF eggs and it was highly significant in comparison to all other housing system. ALT, ECA and ORG eggs showed the highest significant concentration in linoleic acid when compared to LIF eggs. The highest α-linolenic acid concentration was recorded in ECA eggs, a significant lower proportion was found in ALT and ORG eggs, whereas it was not detected in LIF eggs. ORG eggs showed a significantly lower concentration in arachidonic acid compared to the ALT eggs, and ECA and LIF showed an intermediate mean proportion. Docosahexaenoic acid ranged from 1.2 to 1.6% and mean values did not differ significantly between systems. ORG and ALT eggs showed the highest concentration in SFA and it was significantly different compared to the concentration of ECA and LIF eggs. The proportion of MUFA was significantly higher in LIF eggs and similar lower mean values were recorded in the other production systems. The highest PUFA proportion was recorded in ECA eggs and a progressive lower content was recorded in ALT, ORG, and LIF eggs, the latter showing the significant lowest proportion compared to all other system groups. The n-6/n-3 ratio was significantly different between ECA and ALT eggs, showing the lowest (10.4) and highest (16.1) value respectively, and similar intermediate values were calculated in LIF and ORG system (Table 3).

Considering the effect of the OSA, the fatty acid profile in OUT and IND eggs showed some significant differences (Table 3): the proportion of palmitic, stearic and linoleic acids was higher in eggs from outdoor reared hens, on the contrary oleic acid was higher in eggs from indoor reared hens. The total proportion of SFA and PUFA was significantly higher in OUT eggs, whereas the total proportion of MUFA was significantly higher in IND eggs. The n6/n3 ratio was significantly higher in OUT eggs.

**Figure 1:**
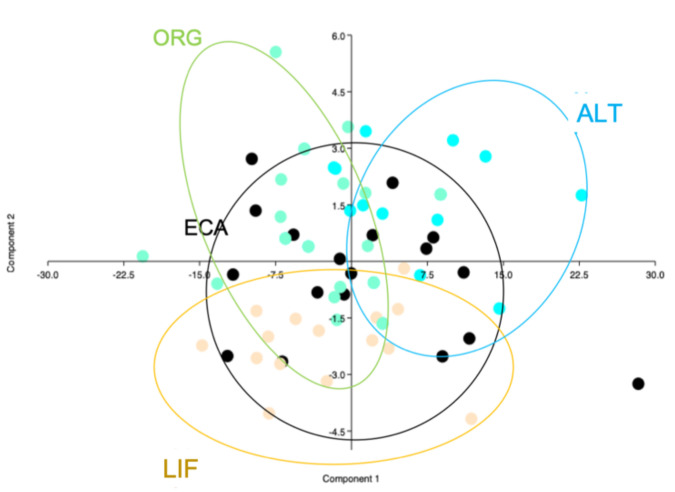
Principal component analysis with physical parameters; component 1 (horizontal axis) and 2 (vertical axis). ALT = alternative with outdoor space, ECA = enriched cage, LIF = litter floor, ORG = organic. IND = only indoor, OUT = outdoor space available.

PCA results with physical parameters is presented in Figure 1.

In physical parameters, components 1 and 2 define the 98% of the variance. In Component one the two leading variables are WEG (0.63) and ALB (0.48). In Component 2, YOL is the most determinant variable (0.69). Wide overlapping areas for ECA eggs with all the other clusters are clearly visible, ORG and ALT eggs are mainly differentiated on Component 1, on the other hand, LIF eggs are principally differentiated on the Component 2 (Tables S1–S3).

**Figure 2:**
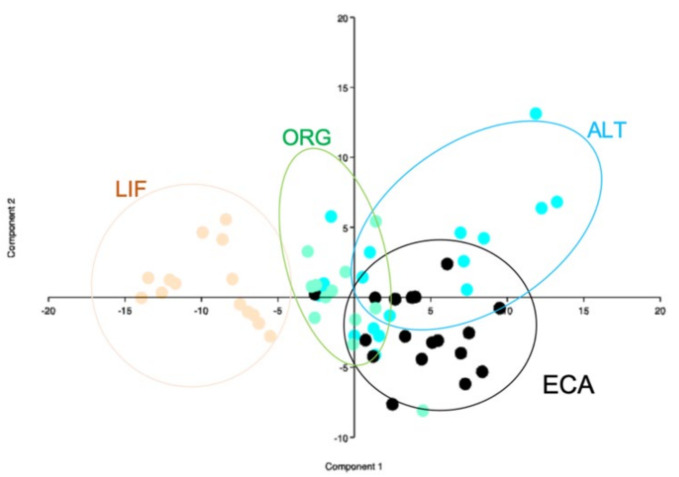
Principal component analysis with fatty acids profile; component 1 (horizontal axis) and 2 (vertical axis). ALT = alternative with outdoor space, ECA enriched cage, LIF = litter floor, ORG = organic. IND = only indoor, OUT = outdoor space available.

PCA results for fatty acids profile are presented in Figure 2. On Component 1, LIF eggs were totally separated from the other systems’ eggs, on the same component ECA eggs showed some overlapping ALT eggs.

In fatty acids the 82% of the variation is defined on the first two components (1 and 2): PUFA and linoleic acid concentrations are the determining variables on Component 1 (0.50 and 0.46). On Component 2 the most influencing parameter is n6/n3 ratio (0.98). LIF eggs are totally separated from the other clusters, ECA eggs show some overlapping with ALT eggs cluster which at the same time is slightly overlapped with the compactly clustered ORG eggs (Tables S4–S6).

## 4. Discussion

In accordance with Taylor [19], who studied eggs produced in cages and aviaries and who found no significant difference in average egg weight between the two housing systems, we did not found any significant difference in IND and OUT eggs’ weight also. Anyway, in our study, that was based on commercial food store marketed eggs, we do not know important parameters determining eggs’ weight like hen genetic strain and age at laying for example. Scientific findings about the weight of eggs produced in different systems are contrasting [20], our results are in accordance to those reported by Minelli and colleagues [21] who found organic eggs laid by the same hybrid at the same age to be the lightest (64.4 vs. 66.2 g) in comparison with those laid in conventional cages. In contrast with our findings (eggshell from ORG system was the lightest), the same authors found no significant differences in eggshell weight between rearing systems. In contrast to finding of Campo and colleagues (2013), we did not find any significant improvement in linolenic acid concentration in free range eggs [22]. Differences between caged and free range hen egg yolk lipids have been described since the late seventies [23]. Egg yolk fatty acid component is mainly affected by feeding programmes [24], dietary lipids, genetics and age [25,26]. However, improvement in housing and husbandry systems may positively influence hens’ welfare, thus improving birds’ life quality and production. In previous researches on table eggs from deep litter, slat floor and mesh floor housing systems no significant differences in egg weight, chemical profiles, and yolk colour were found; on the other hand significant differences were recorded in hygienic aspects and in external egg shell bacterial contamination [27]. The present study confirms the absence of significant differences in eggs’ weight which is a basic parameter in the quality of marketed eggs (2295/2003/EC).

The results show how fatty acid profiles differ in different labelled table eggs. Focusing on fatty acid composition it is important to underline that every considered fatty acid is characterised by different levels in each one of the four labels. ECA and LIF eggs showed the lowest proportion in palmitic acid and stearic acid respectively, on the contrary ORG and ALT eggs recorded the highest content of the same fatty acids. As consequence, OUT eggs had the highest content in palmitic and stearic acids. Our results are in accordance with those of Sammam and colleagues [28] who reported a highest content in stearic acid in organic eggs then in conventional cage system’s eggs (8.77 vs. 8.37%), a higher concentration of palmitic acid too was measured in the organic eggs (25.5 vs. 25.1%), the reported differences were not significant. It has been known for decades that oleic acid is the most represented fatty acid in hen egg; our results are a further confirmation of this trait. Jiang et al. [29] already stressed the hypocholesterolemic effect of oleic acid. In this research the highest concentration of this MUFA has been recorded in LIF eggs and similar values were found in the other productive systems. Indoor reared hens produced eggs with higher oleic acid concentration. Linoleic acid primarily and α-linolenic acid are the essential fatty acids (EFA) for poultry [30]; they play an important role in chick development, and in male reproduction for both their properties and function as precursors of n-6 (linoleic) and n-3 (linolenic) PUFAs. The lowest proportions of linoleic acid have been recorded in LIF eggs, eggs from indoor systems show lower linoleic acid levels compared to eggs produced in outdoor systems. These results are in accordance with Bergami et al. (1978) who found higher level of linoleic acid in non-cage systems [31]. The different concentrations of arachidonic acid must be underlined due to the key role of this fatty acid in prostaglandine metabolism [30]. Comparing the FA contents in hen from a traditional Slovenian breed reared in cage and free range system an increased content in linoleic and arachidonic acids were recorded in cage housed hen (15.07 vs. 12.65%; 2.62 vs. 2.28%); on the contrary α-linolenic acid levels were higher in free range hens (0.65 vs. 0.42%), no statistical significance in differences was calculated [32]. These results are similar to those we described in our groups with or without outdoor space availability (OUT; IND). In the same research a higher content of C20:5n-3 (EPA), C22:6n-3 (DHA), and total n-3 fatty acids have been recorded in free-range hens’ eggs (0.05 vs. 0.01%; 1.47 vs. 1.11%; 2.50 vs. 1.71%); in the cage housed hens higher total PUFA (19.40 vs. 17.43%), in particular higher n-6 PUFA, contents have been described (17.69 vs. 14.93%). According to Jiang et al. (1991) a negative relationship has been calculated between arachidonic acid and longer chain n-3 fatty acids [29]. Sammam and colleagues report a difference in the levels of arachidonic acid in cage eggs compared with barn laid eggs (1.88 vs. 1.83%) [27]; in contrast, our results did not show any significant difference between indoor rearing systems. Anderson in 2011 [33] suggested that the eggs produced in free range facilities to be characterized by higher total fat content and higher MUFA, PUFA, and n-3 PUFA levels in comparison with conventional cages’ eggs (3.80 vs. 3.67%; 1.36 vs. 1.25%, Pastured hens have been described to lay eggs richer in vitamin E and n-3 fatty acids than hens fed only hens’ mash [34]. Krawczyk et al. [35] states a higher content in PUFA and n-3 PUFA in eggs from free range facilities compared with eggs from litter floor facilities, our results confirm the same trend having recorded the highest PUFA content in eggs laid in productive systems providing an outdoor area (0.238 vs. 0.216).

The scatter plots presented in Figure 1 and Figure 2 report the characteristics of the eggs produced in each one of the four considered system to cluster together differentiating from the other systems groups. Good clustering ability has been calculated for fatty acids profile (Figure 1) where overlapping areas are very limited and the four systems are quite differentiated for important nutritional values, such as PUFA content and n6/n3 ratio. Physical parameters show a lower ability in differentiating eggs produced in different systems, anyway some clustering linked to whole egg weight, albumen, and yolk weight has been demonstrated, confirming the importance of easily measurable and perceptible variables in animal product characterization and differentiation.

The present study describes how different label market eggs are characterised by specific profiles in weight parameters (whole egg, yolk, albumen, shell) and their ratios and in yolk fatty acid composition. Hen eggs’ fatty acid levels are strictly linked to birds’ diet formulae [24,36] which heavily influence the nutritive value of the egg. The lack of clear differences in intrinsic egg quality traits within production systems was already described by Yenice et al. [37]. On the contrary Hammershoj [38] found significant differences in fatty acid composition in organic eggs laid by hen reared with open space allowance and grass and herbs availability. In particular, he found higher PUFA concentration, in agreement with the present results, but he described a lower n6/n3 ratio (indoor 11–19 vs. outdoor 5).

## 5. Conclusions

Table eggs physical parameters and fatty acid profile are characterised by high variability within different labelled production systems. Open Space Allowance seems to slightly influence eggs physical parameters and fatty acid profiles. The present results and their comparison with those reported in literature underline the wide differentiation in fatty acid profile and physical parameters in eggs from different production systems. A careful study of the diet to be supplied to hens reared in open air systems and in organic production in particular, considering the strict regulating legislation, could significantly improve the nutritional value of eggs produced in systems focused on hen welfare. Considering the high number of on farm variables influencing nutritional egg quality, a standardized fatty acid profile analysis should be routinely applied on marketed eggs to assess their nutritional value and to increase the number of useful information supplied to consumers. The effects of the complex interactions occurring between production system, hen welfare and eggs quality have been analysed in marketed eggs leading to the conclusion that commercialized market eggs from different production systems are characterized by differences in fatty acid profile and physical parameters. Specific research design limiting influencing factors (genetic, age, diet, climate etc.) should be defined to point out objective differences in physical and fatty acids parameters of eggs produced in different systems.

## Figures and Tables

**Table 1:** Housing characteristics according to Council Directive 1999/74/EC laying down minimum standards for the protection of laying hens; Council Regulation 2007/834/EC and Commission Regulation 2008/889/EC about organic production and labelling of organic products.

Production System	Space Allowance	Indoor Floor	Oudoor Pen
Alternative—ALT	1250 cm^2^/hen	Slat, 15 cm perch/hen, 250 cm^2^ littered area/hen, 1/3 litter	2.5 m^2^/hen
Enriched cage—ECA	750 cm^2^/hen	Wire mesh cage, 15 cm perch/hen	-
Litter floor—LIF	1389 cm^2^/hen	Slat, 250 cm^2^ littered area/hen, 1/3 litter	-
Organic—ORG	1667 cm^2^/hen	Slat, 18 cm perch/hen; 1/3 litter, max 6 hens/m^2^ indoor	4.0 m^2^/hen

**Table 2:** Physical parameters and yolk total lipid in eggs from different housing systems (SYSTEM) and outdoor space allowance (OSA) (LS means ± SE).

Egg Trait ^1^	System				Outdoor Space Allowance	
	ALT	ECA	LIF	ORG	IND	OUT
WE (g)	67.16 ^a^ ± 1.14	62.78 ^b^ ± 1.40	62.11 ^b^ ± 1.25	58.95 ^b^ ± 1.97	62.41 ± 1.03	65.104 ± 1.09
ALB (g)	40.73 ± 0.91	38.98 ± 1.12	39.64 ± 1.001	36.15 ± 1.58	39.35 ± 0.78	39.587 ± 0.826
YOL (g)	17.68 ^a^ ± 0.27	15.47 ^b^ ± 0.34	14.25 ^b^ ± 0.30	15.42 ^b^ ± 0.48	14.79 ^b^ ± 0.27	17.113 ^a^± 0.28
SHE (g)	8.74 ^a^ ± 0.21	8.33 ^a^ ± 0.26	8.23 ^a^ ± 0.23	7.38 ^b^ ± 0.37	8.27 ± 0.19	8.404 ± 0.20
ALB/WE (%)	0.60 ^b^ ± 0.001	0.62 ^ab^ ± 0.01	0.64 ^a^ ± 0.01	0.612 ^ab^ ± 0.01	0.63 ^a^ ± 0.001	0.607 ^b^ ± 0.001
YOLK/WE (%)	0.26 ^a^ ± 0.001	0.27 ^b^ ± 0.001	0.23 ^c^ ± 0.001	0.26 ^ab^ ± 0.01	0.24 ^b^ ± 0.001	0.263 ^a^ ± 0.001
SHE/WE (%)	0.13 ± 0.001	0.13 ± 0.001	0.13 ± 0.001	0.13 ± 0.01	0.13 ± 0.001	0.129 ± 0.001
TL (g/100 g edi)	10.06 ^ac^ ± 0.23	9.53 ^c^ ± 0.29	8.01 ^b^ ± 0.26	11.01 ^a^ ± 0.41	8.69 ^b^ ± 0.22	10.3 ^a^ ± 0.24

^1^ WE = egg weight, ALB = albumen weight, YOL = yolk weight, SHE = shell weight, TL = yolk total lipid. ALT = alternative with outdoor space, ECA = enriched cage, LIF = litter floor, ORG = organic. IND = only indoor, OUT = outdoor space available. Means within a row lacking a common letter differ significantly: a,b,c, (*p* ≤ 0.05).

**Table 3:** Yolk fatty acids concentration (% of total fatty acids; LS Mean ± SE) in eggs from different housing systems and different outdoor space allowance (OSA; IND = indoor, OUT = outdoor space). (LS means ± SE).

Fatty Acid	Housing System				Outdoor Space Allowance	
	ALT	ECA	LIF	ORG	IND	OUT
C16:0	25.7 ^a^ ± 0.2	24.5 ^b^ ± 0.3	24.8 ^ab^ ± 0.3	25.8 ^ab^ ± 0.4	24.6 ^b^ ± 0.2	25.7 ^a^ ± 0.2
C18:0	10.1 ^a^ ± 0.2	9.4 ^ab^ ± 0.2	09.1 ^b^ ± 0.2	10.1 ^a^ ± 0.3	9.2 ^b^ ± 0.1	10.1 ^a^ ± 0.1
C18:1n9	36.0 ^b^ ± 0.4	35.4 ^b^ ± 0.5	42.9 ^a^ ± 0.5	37.9 ^b^ ± 0.7	39.6 ^a^ ± 0.6	36.4 ^b^ ± 0.7
C18:2n6	19.9 ^a^ ± 0.5	20.7 ^a^ ± 0.6	14.3 ^b^ ± 0.6	18.3 ^a^ ± 0.9	17.2 ^b^ ± 0.6	19.5 ^a^ ± 0.7
C18:3n3	0.2 ^bc^ ± 0.1	0.8 ^a^ ± 0.1	0.000 ^c^ ± 0.001	0.4 ^b^ ± 0.1	0.4 ± 0.1	0.3 ± 0.1
C20:4n6	3.0 ^a^ ± 0.1	2.7 ^ab^ ± 0.1	2.7 ^ab^ ± 0.1	2.4 ^b^ ± 0.2	2.7 ± 0.1	2.8 ± 0.1
C22:6n3	1.2 ± 0.1	1.6 ± 0.1	1.2 ± 0.1	1.4 ± 0.2	1.4 ± 0.1	1.3 ± 0.1
SFA	35.7 ^a^ ± 0.3	33.9 ^b^ ± 0.3	3.8 ^b^ ± 0.3	35.9 ^a^ ± 0.5	33.9 ^b^ ± 0.2	35.8 ^a^ ± 0.2
MUFA	36.0 ^b^ ± 0.4	35.4 ^b^ ± 0.5	42.9 ^a^ ± 0.5	37.9 ^b^ ± 0.7	39.6 ^a^ ± 0.6	36.4 ^b^ ± 0.7
PUFA	24.3 ^ab^ ± 0.5	25.8 ^a^ ± 0.6	18.3 ^c^ ± 0.6	22.4 ^b^ ± 0.9	21.6 ^b^ ± 0.7	23.8 ^a^ ± 0.7
n6/n3	16.2 ^a^ ± 0.8	10.4 ^b^ ± 1.0	13.5 ^ab^ ± 0.9	13.9 ^ab^ ± 1.5	12.1 ^b^ ± 0.7	15.5 ^a^ ± 0.7

C16:0 palmitic, C18:0 stearic, C18:1n-9 oleic, C18:2n-6 linoleic, C18:3n-3 linolenic, C20:4n-6 arachidonic, C20:5n-3 EPA eicosapentaenoic, C22:6n-3 DHA docosaexaenoic, SFA = saturated fatty acids, MUFA = monounsaturated fatty acids, PUFA = polyunsaturated fatty acids, ALT = alternative with outdoor space, ECA = enriched cage, LIF = litter floor, ORG = organic. IND = only indoor, OUT = outdoor space available. Means within a row lacking a common letter differ significantly: a,b,c, (*p* ≤ 0.05).

## Supplementary Materials

The following are available online at https://www.mdpi.com/2076-2615/11/2/265/s1, Table S1: PCA results for physical parameters: Eigenvalue, % variance, Table S2: PCA results for physical parametrs: Loadings, Table S3: PCA results for physical parametrs: Scree plot. Eighenvalue %: Explained Variance (%), Table S4: PCA results for fatty acids profile: Eigenvalue, % variance, Table S5: PCA results for fatty acids profile: Loadings, Table S6: PCA results for fatty acids profile: Scree plot. Eighenvalue %: Explained Variance (%).

## References

[1] Grunert K.G. (2005). Food quality and safety: Consumer perception and demand. Eur. Rev. Agric. Econ..

[2] Brunsø K., Fjord T.A., Grunert K.G. (2002). Consumers’ Food Choice and Quality Perception.

[3] Mine Y., Kovacs-Nolan J. (2004). Biologically active hen egg components in human health and disease. J. Poult. Sci..

[4] Nardone A., Valfrè F. (1999). Effects of changing production methods on quality of meat, milk and eggs. Livest. Prod. Sci..

[5] Aumaıtre A. (1999). Quality and safety of animal products. Livest. Prod. Sci..

[6] Spedding C.R.W. (1995). Sustainability in animal production systems. Anim. Sci..

[7] Surai P.F., Sparks N.H.C. (2001). Designer eggs: From improvement of egg composition to functional food. Trends Food Sci. Technol..

[8] Noble R.C., Speake B.K., McCartney R., Foggin C.M., Deeming D.C. (1996). Yolk lipids and their fatty acids in the wild and captive ostrich (Struthio camelus). Comp. Biochem. Physiol. Part B Biochem. Mol. Biol..

[9] Speake B.K., Murray A.M.B., Noble R.C. (1998). Transport and transformations of yolk lipids during development of the avian embryo. Prog. Lipid Res..

[10] Uauy R., Castillo C. (2003). Lipid requirements of infants: Implications for nutrient composition of fortified complementary foods. J. Nutr..

[11] Drakley C., Elson H.A., Walker A.W. Production efficiency of laying hens at four stocking densities in furnished cages of two heights. Proceedings of the 11th European Poultry Conference.

[12] Rushen J., de Passillé A.M.B. (1992). The scientific assessment of the impact of housing on animal welfare: A critical review. Can. J. Anim. Sci..

[13] Christie W.W. (1982). Lipid Analysis.

[14] Folch J., Lees M., Stanley G.H.S. (1957). A simple method for the isolation and purification of total lipides from animal tissues. J. Biol. Chem..

[15] Hamilton R.J., Hamilton S., Harwood J. (1992). Lipid Analysis: A Practical Approach.

[16] Christie W.W., Noble R.C., Moore J.H. (1970). Determination of lipid classes by a gas-chromatographic procedure. Analyst.

[17] (2019). IBM SPSS Statistics software: SPSS Statistics, V26.

[18] Hammer Ø., Harper D.A.T., Ryan P.D. (2001). PAST: Paleontological statistics software package for education and data analysis. Palaeontol. Electron..

[19] Taylor A.A., Hurnik J.F. (1996). The long-term productivity of hens housed in battery cages and an aviary. Poult. Sci..

[20] Rakonjac S., Bogosavljević-Bošković S., Pavlovski Z., Škrbić Z., Dosković V., Petrović M.D., Petričević V. (2014). Laying hen rearing systems: A review of major production results and egg quality traits. Worlds Poult. Sci. J..

[21] Minelli G., Sirri F., Folegatti E., Meluzzi A., Franchini A. (2007). Egg quality traits of laying hens reared in organic and conventional systems. Ital. J. Anim. Sci..

[22] Campo J.L., Cabezas R., Torres O., Briones I.G., Alonso C. (2013). Egg quality and welfare of white-, tinted-, and brown-shell egg layers in three different non-cage housing systems. Arch. Geflugelkd..

[23] Stadelman W.J., Pratt D.E. (1989). Factors influencing composition of the hen’s egg. Worlds Poult. Sci. J..

[24] Milinsk M.C., Murakami A.E., Gomes S.T.M., Matsushita M., De Souza N.E. (2003). Fatty acid profile of egg yolk lipids from hens fed diets rich in n-3 fatty acids. Food Chem..

[25] Cobos A., de la Hoz L., Cambero M.I., Ordóñez J.A. (1995). Dietary modification and hen strain dependence of egg yolk lipids. Food Res. Int..

[26] Marelli S.P., Zaniboni L., Madeddu M., Sayed A.A., Strillacci M.G., Mangiagalli M.G., Cerolini S. (2020). Physical Parameters and Fatty Acids Profiles in Milanino, Mericanel Della Brianza, Valdarnese Bianca and Commercial Hybrids (*Gallus Gallus Domesticus*) Table Eggs. Animals.

[27] Huneau-Salaün A., Michel V., Huonnic D., Balaine L., Le Bouquin S. (2010). Quality of eggs from Polish native Greenleg Partridge chicken-hens maintained in organic vs. backyard production system. Br. Poult. Sci..

[28] Samman S., Kung F.P., Carter L.M., Foster M.J., Ahmad Z.I., Phuyal J.L., Petocz P. (2009). Fatty acid composition of certified organic, conventional and omega-3 eggs. Food Chem..

[29] Jiang Z., Ahn D.U., Sim J.S. (1991). Effects of feeding flax and two types of sunflower seeds on fatty acid compositions of yolk lipid classes. Poult. Sci..

[30] Watkins B.A. (1995). Biochemical and physiological aspects of polyunsaturates. Poult. Avian Biol. Rev..

[31] Bergami W., Giavarini I., Minoccheri F., Negrini F. (1978). Egg composition and breeding technology. Fat contents of the eggs laid by chickens bred in battery or free range. Avicoltura.

[32] Simčič M., Stibilj V., Holcman A. (2011). Fatty acid composition of eggs produced by the Slovenian autochthonous Styrian hen. Food Chem..

[33] Anderson K.E. (2011). Comparison of fatty acid, cholesterol, and vitamin A and E composition in eggs from hens housed in conventional cage and range production facilities. Poult. Sci..

[34] Karsten H.D., Patterson P.H., Stout R., Crews G. (2010). Vitamins A, E and fatty acid composition of the eggs of caged hens and pastured hens. Renew. Agric. Food Syst..

[35] Krawczyk J., Sokołowicz Z., Szymczyk B. (2011). Effect of housing system on cholesterol, vitamin and fatty acid content of yolk and physical characteristics of eggs from Polish native hens. Arch. Geflügelkd.

[36] Zaniboni L., La Cognata R., Cerolini S. (2006). Qualità dell’uovo da consumo nei diversi sistemi di allevamento considerati dalla normativa in vigore. Riv. Avic..

[37] Yenice G., Kaynar O., Ileriturk M., Hira F., Hayirli A. (2016). Quality of eggs in different production systems. Czech J. Food Sci..

[38] Hammershøj M., Johansen N.F. (2016). The effect of grass and herbs in organic egg production on egg fatty acid composition, egg yolk colour and sensory properties. Livest. Sci..

