# Supplementary material for: Egg Production Systems, Open Space Allowance and Their Effects on Physical Parameters and Fatty Acid Profile in Commercial Eggs
Source: Animals (Basel). 2021 Jan 21;11(2):265. doi: 10.3390/ani11020265 (PMC7911268; doi:10.3390/ani11020265)

Add. Tab. 1 PCA results for physical parametrs: Eigenvalue, % variance

PC	Eigenvalue	% variance
1	72.6849	93.41
2	4.20123	5.3992
3	0.926531	1.1907
4	1.54974E-05	1.9916E-05
5	9.00949E-06	1.1578E-05
6	7.62597E-06	9.8004E-06
7	2.5821E-29	3.3183E-29
8	3.12624E-30	4.0177E-30

Add. Tab. 2 PCA results for physical parametrs: Loadings

	PC 1	PC 2
we	0.63639	0.27956
alb	0.48033	-0.62767
yol	0.1112	0.6904
she	0.044858	0.21684
edi	0.59153	0.062725
albwe	0.0013325	-0.012644
yolwe	-0.00064523	0.0097607
shewe	-0.00057403	0.002885

Add. Tab. 3 PCA results for physical parametrs: Scree plot. Eighenvalue %: Explained Variance (%)

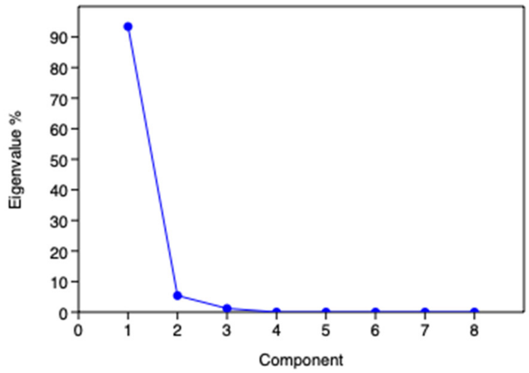

Add. Tab. 4 PCA results for fatty acids profile: Eigenvalue, % variance

PC	Eigenvalue	% variance
1	42.8151	62.001
2	13.5377	19.604
3	7.79607	11.29
4	3.81946	5.531
5	0.613785	0.88883
6	0.315414	0.45675
7	0.132915	0.19248
8	0.0251386	0.036403
9	2.45218E-05	3.551E-05
10	1.21215E-05	1.7553E-05
11	5.66274E-32	8.2003E-32

Add. Tab. 5 PCA results for fatty acids profile: Loadings

	PC 1	PC 2
C16:0	-0.01782	-0.012387
C18:0	0.037508	0.0033927
C18:1n9	-0.51389	0.11537
C18:2n6	0.46304	0.10376
C18:3n3	0.020023	-0.056158
C20:4n6	0.0075267	-0.0051382
C22:6n3	0.0067876	-0.060343
SFA	0.019626	-0.0088739
MUFA	-0.51389	0.11537
PUFA	0.49779	-0.019909
n6/n3	0.083747	0.97732

Add. Tab. 6 PCA results for fatty acids profile: Scree plot. Eighenvalue %: Explained Variance (%)

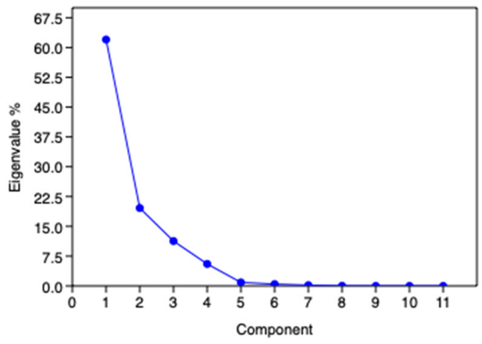

Supplement: Supplementary file 1 [file animals-11-00265-s001.pdf]
